# Supplementary material for: Pyruvate Dehydrogenase Kinase 1 inhibition mediated oxidative phosphorylation enhancement in cartilage promotes osteoarthritis progression
Source: BMC Musculoskelet Disord. 2023 Jul 20;24:597. doi: 10.1186/s12891-023-06585-6 (PMC10357736; doi:10.1186/s12891-023-06585-6)
Supplement: Supplementary file 1 — Additional file 1. [file 12891_2023_6585_MOESM1_ESM.zip › MF.Gsea.1653627316242/GOMF_HISTONE_DEACETYLASE_BINDING.html]

Details for gene set GOMF\_HISTONE\_DEACETYLASE\_BINDING[GSEA]

|  || Dataset | 55457\_collapsed\_to\_symbols.cls.cls #OA\_versus\_normal.cls.cls #OA\_versus\_normal\_repos |
| Phenotype | cls.cls#OA\_versus\_normal\_repos |
| Upregulated in class | normal |
| GeneSet | GOMF\_HISTONE\_DEACETYLASE\_BINDING |
| Enrichment Score (ES) | -0.3289974 |
| Normalized Enrichment Score (NES) | -1.439615 |
| Nominal p-value | 0.0171875 |
| FDR q-value | 0.2413275 |
| FWER p-Value | 1.0 |
Table: GSEA Results Summary

  

Fig 1: Enrichment plot: GOMF\_HISTONE\_DEACETYLASE\_BINDING      
 Profile of the Running ES Score & Positions of GeneSet Members on the Rank Ordered List

  

| SYMBOL | TITLE | RANK IN GENE LIST | RANK METRIC SCORE | RUNNING ES | CORE ENRICHMENT || 1 | BEX4 | brain expressed X-linked 4 [Source:HGNC Symbol;Acc:HGNC:25475] | 113 | 0.896 | 0.0191 | No |
| 2 | BHLHE41 | basic helix-loop-helix family member e41 [Source:HGNC Symbol;Acc:HGNC:16617] | 417 | 0.700 | 0.0172 | No |
| 3 | CDC20 | cell division cycle 20 [Source:HGNC Symbol;Acc:HGNC:1723] | 552 | 0.641 | 0.0266 | No |
| 4 | RBBP4 | "RB binding protein 4, chromatin remodeling factor [Source:HGNC Symbol;Acc:HGNC:9887]" | 1181 | 0.480 | -0.0075 | No |
| 5 | HDAC3 | histone deacetylase 3 [Source:HGNC Symbol;Acc:HGNC:4854] | 1325 | 0.454 | -0.0046 | No |
| 6 | RFXANK | regulatory factor X associated ankyrin containing protein [Source:HGNC Symbol;Acc:HGNC:9987] | 1383 | 0.445 | 0.0048 | No |
| 7 | NUDT21 | nudix hydrolase 21 [Source:HGNC Symbol;Acc:HGNC:13870] | 1734 | 0.393 | -0.0103 | No |
| 8 | PRKN | parkin RBR E3 ubiquitin protein ligase [Source:HGNC Symbol;Acc:HGNC:8607] | 1972 | 0.364 | -0.0175 | No |
| 9 | HOXA10 | homeobox A10 [Source:HGNC Symbol;Acc:HGNC:5100] | 2408 | 0.313 | -0.0417 | No |
| 10 | H1-5 | "H1.5 linker histone, cluster member [Source:HGNC Symbol;Acc:HGNC:4719]" | 2518 | 0.301 | -0.0408 | No |
| 11 | INSM1 | INSM transcriptional repressor 1 [Source:HGNC Symbol;Acc:HGNC:6090] | 2531 | 0.299 | -0.0325 | No |
| 12 | PKN1 | protein kinase N1 [Source:HGNC Symbol;Acc:HGNC:9405] | 2540 | 0.298 | -0.0238 | No |
| 13 | NCOR1 | nuclear receptor corepressor 1 [Source:HGNC Symbol;Acc:HGNC:7672] | 2549 | 0.297 | -0.0152 | No |
| 14 | NACC2 | NACC family member 2 [Source:HGNC Symbol;Acc:HGNC:23846] | 2655 | 0.284 | -0.0145 | No |
| 15 | GLI3 | GLI family zinc finger 3 [Source:HGNC Symbol;Acc:HGNC:4319] | 2861 | 0.264 | -0.0223 | No |
| 16 | BCOR | BCL6 corepressor [Source:HGNC Symbol;Acc:HGNC:20893] | 2890 | 0.262 | -0.0164 | No |
| 17 | MAGEA2 | MAGE family member A2 [Source:HGNC Symbol;Acc:HGNC:6800] | 2952 | 0.255 | -0.0132 | No |
| 18 | MAGEA3 | MAGE family member A3 [Source:HGNC Symbol;Acc:HGNC:6801] | 3034 | 0.246 | -0.0119 | No |
| 19 | MAGEA1 | MAGE family member A1 [Source:HGNC Symbol;Acc:HGNC:6796] | 3105 | 0.239 | -0.0099 | No |
| 20 | DACT1 | dishevelled binding antagonist of beta catenin 1 [Source:HGNC Symbol;Acc:HGNC:17748] | 3275 | 0.223 | -0.0162 | No |
| 21 | PARP1 | poly(ADP-ribose) polymerase 1 [Source:HGNC Symbol;Acc:HGNC:270] | 3410 | 0.210 | -0.0201 | No |
| 22 | TOP2B | DNA topoisomerase II beta [Source:HGNC Symbol;Acc:HGNC:11990] | 3458 | 0.206 | -0.0174 | No |
| 23 | RELA | "RELA proto-oncogene, NF-kB subunit [Source:HGNC Symbol;Acc:HGNC:9955]" | 3942 | 0.161 | -0.0501 | No |
| 24 | HEY2 | hes related family bHLH transcription factor with YRPW motif 2 [Source:HGNC Symbol;Acc:HGNC:4881] | 4058 | 0.150 | -0.0544 | No |
| 25 | WDTC1 | WD and tetratricopeptide repeats 1 [Source:HGNC Symbol;Acc:HGNC:29175] | 4121 | 0.144 | -0.0548 | No |
| 26 | MEF2C | myocyte enhancer factor 2C [Source:HGNC Symbol;Acc:HGNC:6996] | 4218 | 0.135 | -0.0581 | No |
| 27 | TCF21 | transcription factor 21 [Source:HGNC Symbol;Acc:HGNC:11632] | 4321 | 0.127 | -0.0621 | No |
| 28 | HDAC5 | histone deacetylase 5 [Source:HGNC Symbol;Acc:HGNC:14068] | 4336 | 0.125 | -0.0593 | No |
| 29 | MAGEA10 | MAGE family member A10 [Source:HGNC Symbol;Acc:HGNC:6797] | 4360 | 0.124 | -0.0572 | No |
| 30 | HNRNPD | heterogeneous nuclear ribonucleoprotein D [Source:HGNC Symbol;Acc:HGNC:5036] | 4830 | 0.085 | -0.0913 | No |
| 31 | MAGEA8 | MAGE family member A8 [Source:HGNC Symbol;Acc:HGNC:6806] | 4846 | 0.084 | -0.0898 | No |
| 32 | KAT2B | lysine acetyltransferase 2B [Source:HGNC Symbol;Acc:HGNC:8638] | 4867 | 0.082 | -0.0888 | No |
| 33 | CBX5 | chromobox 5 [Source:HGNC Symbol;Acc:HGNC:1555] | 4963 | 0.075 | -0.0939 | No |
| 34 | PHB | prohibitin [Source:HGNC Symbol;Acc:HGNC:8912] | 5102 | 0.064 | -0.1027 | No |
| 35 | HDAC9 | histone deacetylase 9 [Source:HGNC Symbol;Acc:HGNC:14065] | 5183 | 0.058 | -0.1072 | No |
| 36 | TAL1 | "TAL bHLH transcription factor 1, erythroid differentiation factor [Source:HGNC Symbol;Acc:HGNC:11556]" | 5406 | 0.040 | -0.1233 | No |
| 37 | TP53 | tumor protein p53 [Source:HGNC Symbol;Acc:HGNC:11998] | 5511 | 0.032 | -0.1304 | No |
| 38 | MAGEA4 | MAGE family member A4 [Source:HGNC Symbol;Acc:HGNC:6802] | 5553 | 0.028 | -0.1327 | No |
| 39 | MEF2A | myocyte enhancer factor 2A [Source:HGNC Symbol;Acc:HGNC:6993] | 5628 | 0.022 | -0.1379 | No |
| 40 | TRAF6 | TNF receptor associated factor 6 [Source:HGNC Symbol;Acc:HGNC:12036] | 5761 | 0.011 | -0.1478 | No |
| 41 | ANKRA2 | ankyrin repeat family A member 2 [Source:HGNC Symbol;Acc:HGNC:13208] | 5840 | 0.006 | -0.1537 | No |
| 42 | SIRT2 | sirtuin 2 [Source:HGNC Symbol;Acc:HGNC:10886] | 6279 | -0.026 | -0.1872 | No |
| 43 | TOP2A | DNA topoisomerase II alpha [Source:HGNC Symbol;Acc:HGNC:11989] | 6377 | -0.034 | -0.1937 | No |
| 44 | THAP7 | THAP domain containing 7 [Source:HGNC Symbol;Acc:HGNC:23190] | 6412 | -0.036 | -0.1952 | No |
| 45 | ANKRD1 | ankyrin repeat domain 1 [Source:HGNC Symbol;Acc:HGNC:15819] | 6416 | -0.037 | -0.1943 | No |
| 46 | MAGEA12 | MAGE family member A12 [Source:HGNC Symbol;Acc:HGNC:6799] | 6439 | -0.038 | -0.1949 | No |
| 47 | NCOR2 | nuclear receptor corepressor 2 [Source:HGNC Symbol;Acc:HGNC:7673] | 6497 | -0.042 | -0.1980 | No |
| 48 | BRMS1 | BRMS1 transcriptional repressor and anoikis regulator [Source:HGNC Symbol;Acc:HGNC:17262] | 6602 | -0.050 | -0.2046 | No |
| 49 | TFAP4 | transcription factor AP-4 [Source:HGNC Symbol;Acc:HGNC:11745] | 6823 | -0.065 | -0.2198 | No |
| 50 | IKZF3 | IKAROS family zinc finger 3 [Source:HGNC Symbol;Acc:HGNC:13178] | 6905 | -0.073 | -0.2238 | No |
| 51 | FOXP3 | forkhead box P3 [Source:HGNC Symbol;Acc:HGNC:6106] | 7035 | -0.082 | -0.2314 | No |
| 52 | BORCS8-MEF2B | BORCS8-MEF2B readthrough [Source:HGNC Symbol;Acc:HGNC:39979] | 7093 | -0.087 | -0.2331 | No |
| 53 | SP2 | Sp2 transcription factor [Source:HGNC Symbol;Acc:HGNC:11207] | 7209 | -0.097 | -0.2391 | No |
| 54 | LEF1 | lymphoid enhancer binding factor 1 [Source:HGNC Symbol;Acc:HGNC:6551] | 7467 | -0.116 | -0.2556 | No |
| 55 | CCND1 | cyclin D1 [Source:HGNC Symbol;Acc:HGNC:1582] | 7512 | -0.119 | -0.2553 | No |
| 56 | NR2E1 | nuclear receptor subfamily 2 group E member 1 [Source:HGNC Symbol;Acc:HGNC:7973] | 7660 | -0.132 | -0.2627 | No |
| 57 | HDAC6 | histone deacetylase 6 [Source:HGNC Symbol;Acc:HGNC:14064] | 7833 | -0.143 | -0.2717 | No |
| 58 | GMNN | geminin DNA replication inhibitor [Source:HGNC Symbol;Acc:HGNC:17493] | 7866 | -0.146 | -0.2696 | No |
| 59 | HSPA1A | heat shock protein family A (Hsp70) member 1A [Source:HGNC Symbol;Acc:HGNC:5232] | 7876 | -0.146 | -0.2658 | No |
| 60 | HDAC1 | histone deacetylase 1 [Source:HGNC Symbol;Acc:HGNC:4852] | 7911 | -0.148 | -0.2638 | No |
| 61 | HDAC4 | histone deacetylase 4 [Source:HGNC Symbol;Acc:HGNC:14063] | 8416 | -0.187 | -0.2974 | No |
| 62 | NRIP1 | nuclear receptor interacting protein 1 [Source:HGNC Symbol;Acc:HGNC:8001] | 8524 | -0.195 | -0.2997 | No |
| 63 | AKAP8 | A-kinase anchoring protein 8 [Source:HGNC Symbol;Acc:HGNC:378] | 8659 | -0.205 | -0.3038 | No |
| 64 | ZBTB7B | zinc finger and BTB domain containing 7B [Source:HGNC Symbol;Acc:HGNC:18668] | 8702 | -0.209 | -0.3005 | No |
| 65 | CHD4 | chromodomain helicase DNA binding protein 4 [Source:HGNC Symbol;Acc:HGNC:1919] | 8917 | -0.226 | -0.3102 | No |
| 66 | NR2C1 | nuclear receptor subfamily 2 group C member 1 [Source:HGNC Symbol;Acc:HGNC:7971] | 8940 | -0.227 | -0.3049 | No |
| 67 | MAPK8 | mitogen-activated protein kinase 8 [Source:HGNC Symbol;Acc:HGNC:6881] | 8956 | -0.228 | -0.2990 | No |
| 68 | SPI1 | Spi-1 proto-oncogene [Source:HGNC Symbol;Acc:HGNC:11241] | 9012 | -0.232 | -0.2960 | No |
| 69 | LCOR | ligand dependent nuclear receptor corepressor [Source:HGNC Symbol;Acc:HGNC:29503] | 9227 | -0.250 | -0.3050 | No |
| 70 | HDAC2 | histone deacetylase 2 [Source:HGNC Symbol;Acc:HGNC:4853] | 9359 | -0.260 | -0.3071 | No |
| 71 | CIR1 | "corepressor interacting with RBPJ, CIR1 [Source:HGNC Symbol;Acc:HGNC:24217]" | 9640 | -0.287 | -0.3201 | Yes |
| 72 | SFPQ | splicing factor proline and glutamine rich [Source:HGNC Symbol;Acc:HGNC:10774] | 9747 | -0.296 | -0.3191 | Yes |
| 73 | KPNA2 | karyopherin subunit alpha 2 [Source:HGNC Symbol;Acc:HGNC:6395] | 9753 | -0.297 | -0.3103 | Yes |
| 74 | RAD9A | RAD9 checkpoint clamp component A [Source:HGNC Symbol;Acc:HGNC:9827] | 9757 | -0.297 | -0.3013 | Yes |
| 75 | KAT2A | lysine acetyltransferase 2A [Source:HGNC Symbol;Acc:HGNC:4201] | 9799 | -0.301 | -0.2951 | Yes |
| 76 | SATB2 | SATB homeobox 2 [Source:HGNC Symbol;Acc:HGNC:21637] | 9883 | -0.310 | -0.2920 | Yes |
| 77 | HSP90AB1 | heat shock protein 90 alpha family class B member 1 [Source:HGNC Symbol;Acc:HGNC:5258] | 10060 | -0.327 | -0.2956 | Yes |
| 78 | RARA | retinoic acid receptor alpha [Source:HGNC Symbol;Acc:HGNC:9864] | 10141 | -0.335 | -0.2914 | Yes |
| 79 | SMG5 | SMG5 nonsense mediated mRNA decay factor [Source:HGNC Symbol;Acc:HGNC:24644] | 10190 | -0.340 | -0.2846 | Yes |
| 80 | GCM1 | glial cells missing transcription factor 1 [Source:HGNC Symbol;Acc:HGNC:4197] | 10236 | -0.345 | -0.2773 | Yes |
| 81 | PRDM1 | PR/SET domain 1 [Source:HGNC Symbol;Acc:HGNC:9346] | 10374 | -0.358 | -0.2769 | Yes |
| 82 | MAGEA11 | MAGE family member A11 [Source:HGNC Symbol;Acc:HGNC:6798] | 10458 | -0.369 | -0.2719 | Yes |
| 83 | MAGEA5P | "MAGE family member A5, pseudogene [Source:HGNC Symbol;Acc:HGNC:6803]" | 10577 | -0.381 | -0.2693 | Yes |
| 84 | MTA2 | metastasis associated 1 family member 2 [Source:HGNC Symbol;Acc:HGNC:7411] | 10600 | -0.383 | -0.2590 | Yes |
| 85 | MEF2D | myocyte enhancer factor 2D [Source:HGNC Symbol;Acc:HGNC:6997] | 11149 | -0.451 | -0.2878 | Yes |
| 86 | CRY1 | cryptochrome circadian regulator 1 [Source:HGNC Symbol;Acc:HGNC:2384] | 11247 | -0.463 | -0.2810 | Yes |
| 87 | NKX3-1 | NK3 homeobox 1 [Source:HGNC Symbol;Acc:HGNC:7838] | 11348 | -0.478 | -0.2739 | Yes |
| 88 | MIER2 | MIER family member 2 [Source:HGNC Symbol;Acc:HGNC:29210] | 11375 | -0.483 | -0.2610 | Yes |
| 89 | BCL3 | BCL3 transcription coactivator [Source:HGNC Symbol;Acc:HGNC:998] | 11431 | -0.493 | -0.2499 | Yes |
| 90 | YWHAB | tyrosine 3-monooxygenase/tryptophan 5-monooxygenase activation protein beta [Source:HGNC Symbol;Acc:HGNC:12849] | 11488 | -0.500 | -0.2387 | Yes |
| 91 | TBX2 | T-box transcription factor 2 [Source:HGNC Symbol;Acc:HGNC:11597] | 11728 | -0.538 | -0.2407 | Yes |
| 92 | HIF1A | hypoxia inducible factor 1 subunit alpha [Source:HGNC Symbol;Acc:HGNC:4910] | 11833 | -0.556 | -0.2315 | Yes |
| 93 | HIC1 | HIC ZBTB transcriptional repressor 1 [Source:HGNC Symbol;Acc:HGNC:4909] | 12118 | -0.624 | -0.2343 | Yes |
| 94 | YWHAE | tyrosine 3-monooxygenase/tryptophan 5-monooxygenase activation protein epsilon [Source:HGNC Symbol;Acc:HGNC:12851] | 12137 | -0.628 | -0.2161 | Yes |
| 95 | PKN2 | protein kinase N2 [Source:HGNC Symbol;Acc:HGNC:9406] | 12237 | -0.655 | -0.2035 | Yes |
| 96 | HSPA1B | heat shock protein family A (Hsp70) member 1B [Source:HGNC Symbol;Acc:HGNC:5233] | 12278 | -0.670 | -0.1858 | Yes |
| 97 | NIPBL | NIPBL cohesin loading factor [Source:HGNC Symbol;Acc:HGNC:28862] | 12385 | -0.703 | -0.1722 | Yes |
| 98 | MAGEA9 | MAGE family member A9 [Source:HGNC Symbol;Acc:HGNC:6807] | 12513 | -0.751 | -0.1587 | Yes |
| 99 | SRF | serum response factor [Source:HGNC Symbol;Acc:HGNC:11291] | 12549 | -0.773 | -0.1374 | Yes |
| 100 | SP1 | Sp1 transcription factor [Source:HGNC Symbol;Acc:HGNC:11205] | 12557 | -0.777 | -0.1137 | Yes |
| 101 | AKAP8L | A-kinase anchoring protein 8 like [Source:HGNC Symbol;Acc:HGNC:29857] | 12665 | -0.836 | -0.0961 | Yes |
| 102 | HES1 | hes family bHLH transcription factor 1 [Source:HGNC Symbol;Acc:HGNC:5192] | 12676 | -0.849 | -0.0705 | Yes |
| 103 | HSP90AA1 | heat shock protein 90 alpha family class A member 1 [Source:HGNC Symbol;Acc:HGNC:5253] | 12730 | -0.893 | -0.0468 | Yes |
| 104 | MTA1 | metastasis associated 1 [Source:HGNC Symbol;Acc:HGNC:7410] | 12792 | -0.952 | -0.0219 | Yes |
| 105 | CEBPB | CCAAT enhancer binding protein beta [Source:HGNC Symbol;Acc:HGNC:1834] | 12804 | -0.973 | 0.0075 | Yes |
Table: GSEA details [plain text format]

  

Fig 2: GOMF\_HISTONE\_DEACETYLASE\_BINDING      
 Blue-Pink O' Gram in the Space of the Analyzed GeneSet

  

Fig 3: GOMF\_HISTONE\_DEACETYLASE\_BINDING: Random ES distribution      
 Gene set null distribution of ES for **GOMF\_HISTONE\_DEACETYLASE\_BINDING**
